# Suitable Electrode Choice for Robotic-Assisted Cochlear Implant Surgery: A Systematic Literature Review of Manual Electrode Insertion Adverse Events

**DOI:** 10.3389/fsurg.2022.823219

**Published:** 2022-03-24

**Authors:** Paul Van de Heyning, Peter Roland, Luis Lassaletta, Sumit Agrawal, Marcus Atlas, Wolf-Dieter Baumgartner, Kevin Brown, Marco Caversaccio, Stefan Dazert, Wolfgang Gstoettner, Rudolf Hagen, Abdulrahman Hagr, Greg Eigner Jablonski, Mohan Kameswaran, Vladislav Kuzovkov, Martin Leinung, Yongxin Li, Andreas Loth, Astrid Magele, Robert Mlynski, Joachim Mueller, Lorne Parnes, Andreas Radeloff, Chris Raine, Gunesh Rajan, Joachim Schmutzhard, Henryk Skarzynski, Piotr H. Skarzynski, Georg Sprinzl, Hinrich Staecker, Timo Stöver, Dayse Tavora-Viera, Vedat Topsakal, Shin-Ichi Usami, Vincent Van Rompaey, Nora M. Weiss, Wilhelm Wimmer, Mario Zernotti, Javier Gavilan

**Affiliations:** ^1^Department of Otorhinolaryngology Head and Neck Surgery, Antwerp University Hospital, University of Antwerp, Antwerp, Belgium; ^2^Department of Translational Neurosciences, University of Antwerp, Antwerp, Belgium; ^3^Department of Otolaryngology, Head & Neck Surgery, University of Texas Southwestern Medical Center, Dallas, TX, United States; ^4^Hospital Universitario La Paz, Institute for Health Research (IdiPAZ), Madrid, Spain; ^5^Department of Otolaryngology-Head and Neck Surgery, Western University, London, ON, Canada; ^6^Ear Sciences Institute Australia, Lions Hearing Clinic, Perth, WA, Australia; ^7^Vienna Medical University-General Hospital AKH, Vienna, Austria; ^8^UNC Ear and Hearing Center at Chapel Hill School of Medicine, Chapel Hill, NC, United States; ^9^Department for ENT, Head and Neck Surgery, Bern University Hospital, Bern, Switzerland; ^10^Department of Otorhinolaryngology-Head and Neck Surgery, Ruhr-University Bochum, St. Elisabeth University Hospital Bochum, Bochum, Germany; ^11^Würzburg ENT University Hospital, Würzburg, Germany; ^12^King Abdullah Ear Specialist Center, King Saud University Medical City, King Saud University, Riyadh, Saudi Arabia; ^13^Institute of Clinical Medicine, University of Oslo, Oslo, Norway; ^14^Department of Otorhinolaryngology & Head and Neck Surgery, Oslo University Hospital, Rikshospitalet, Oslo, Norway; ^15^Madras ENT Research Foundation (Pvt) Ltd., Chennai, India; ^16^St. Petersburg ENT and Speech Research Institute, St. Petersburg, Russia; ^17^Department of Otolaryngology, Head and Neck Surgery, University Hospital Frankfurt, Frankfurt am Main, Germany; ^18^Department of Otorhinolaryngology Head and Neck Surgery, Beijing Tongren Hospital, Capital Medical University, Beijing, China; ^19^Key Laboratory of Otolaryngology Head and Neck Surgery (Capital Medical University), Ministry of Education, Beijing, China; ^20^Ear, Nose and Throat Department, University Clinic St. Poelten, Karl Landsteiner Private University, St. Poelten, Austria; ^21^Department of Otorhinolaryngology, Head and Neck Surgery, “Otto Körner” Rostock University Medical Center, Rostock, Germany; ^22^Klinik und Poliklinik für Hals-, Nasen- und Ohrenheilkunde, Ludwig-Maximilians-Universitat Munchen, Munchen, Germany; ^23^Division of Oto-Rhino-Laryngology, Evangelisches Krankenhaus Oldenburg, Research Center of Neurosensory Sciences, University Oldenburg, Oldenburg, Germany; ^24^Bradford Royal Infirmary Yorkshire Auditory Implant Center, Bradford, United Kingdom; ^25^Department of Otolaryngology, Head and Neck Surgery, Luzerner Kantonsspital, Luzern, Medical Sciences Department of Health Sciences and Medicine. University of Lucerne, Luzern, Switzerland. Otolaryngology, Head & Neck Surgery, Medical School University of Western Australia, Perth, WA, Australia; ^26^Department of Otorhinolaryngology, Medical University of Innsbruck, Innsbruck, Austria; ^27^Department of Teleaudiology and Screening, World Hearing Center of the Institute of Physiology and Pathology of Hearing, Kajetany, Poland; ^28^Kansas University Center for Hearing and Balance Disorders, Kansas City, KS, United States; ^29^Fiona Stanley Fremantle Hospitals Group, Perth, WA, Australia; ^30^Department of ENT HNS, University Hospital Brussels, Brussels, Belgium; ^31^Department of Hearing Implant Sciences, Shinshu University School of Medicine, Nagano, Japan; ^32^Catholic University of Córdoba and National University of Córdoba, Córdoba, Argentina

**Keywords:** robotic assisted cochlear implant surgery, pre-shaped electrode, straight electrode, tip fold-over, scalar deviation, electrode migration

## Abstract

**Background and Objective:**

The cochlear implant (CI) electrode insertion process is a key step in CI surgery. One of the aims of advances in robotic-assisted CI surgery (RACIS) is to realize better cochlear structure preservation and to precisely control insertion. The aim of this literature review is to gain insight into electrode selection for RACIS by acquiring a thorough knowledge of electrode insertion and related complications from classic CI surgery involving a manual electrode insertion process.

**Methods:**

A systematic electronic search of the literature was carried out using PubMed, Scopus, Cochrane, and Web of Science to find relevant literature on electrode tip fold over (ETFO), electrode scalar deviation (ESD), and electrode migration (EM) from both pre-shaped and straight electrode types.

**Results:**

A total of 82 studies that include 8,603 ears implanted with a CI, i.e., pre-shaped (4,869) and straight electrodes (3,734), were evaluated. The rate of ETFO (25 studies, 2,335 ears), ESD (39 studies, 3,073 ears), and EM (18 studies, 3,195 ears) was determined. An incidence rate (±95% CI) of 5.38% (4.4–6.6%) of ETFO, 28.6% (26.6–30.6%) of ESD, and 0.53% (0.2–1.1%) of EM is associated with pre-shaped electrodes, whereas with straight electrodes it was 0.51% (0.1–1.3%), 11% (9.2–13.0%), and 3.2% (2.5–3.95%), respectively. The differences between the pre-shaped and straight electrode types are highly significant (*p* < 0.001). Laboratory experiments show evidence that robotic insertions of electrodes are less traumatic than manual insertions. The influence of round window (RW) vs. cochleostomy (Coch) was not assessed.

**Conclusion:**

Considering the current electrode designs available and the reported incidence of insertion complications, the use of straight electrodes in RACIS and conventional CI surgery (and manual insertion) appears to be less traumatic to intracochlear structures compared with pre-shaped electrodes. However, EM of straight electrodes should be anticipated. RACIS has the potential to reduce these complications.

## Introduction

Cochlear implants (CIs) are widely accepted as the *state-of-the-art* hearing solution for *partial-to-profound* sensorineural hearing loss (SNHL) in adults (1) and children (2). The implant's stimulator-receiver is surgically placed under the skin and rests on the surface of the skull. While the electrode array is placed within the cochlea, the excess electrode lead is left coiled in the surgically drilled mastoid cavity (3). The speech processor converts the acoustical signals into electrical signals and is worn externally. The maximum benefit for patients is expected when the electrode array is optimally placed fully inside scala tympani (ST) (or even in scala vestibuli (SV) in special cases of ST ossification) without any degree of scalar deviation, so as to create an effective electrode-neural interface (4).

Intra-cochlear electrode insertion is considered one of the crucial steps of a successful CI surgery. In particular, studies have suggested that slow steady insertion (achieved more easily with robotic insertion) can reduce pressure changes within the cochlea (5, 6), reduce insertion forces (7), and increase the likelihood of an in-axis insertion into ST and improve hearing outcomes (8). Robotic-assisted cochlear implant surgery (RACIS) aims to optimize this insertion process by (1) computer control of insertion speed and by applying insertion forces more steadily and smoothly, (2) defining the angle with which the electrode is inserted into the ST, and (3) improving the estimated insertion depth to minimize trauma and provide better hearing outcomes.

Robotic-assisted cochlear implant surgery has the potential of being included in the surgical armamentarium in the future. Before RACIS can become the standard approach for cochlear implantation, aspects of clinical benefits, cost, and duration of the procedure still need to be addressed (9). Currently, there are three such systems with either Conformité Européenne (CE) or Food and Drug Administration (FDA) approval and a new system is under clinical trial. RobOtol® is a French innovation that recently received the CE mark (10) and the iotaSOFT® insertion system received the American FDA approval in October 2021 (11). These two systems offer automated electrode insertion support after manual drilling of the temporal bone to reach the round window (RW) niche. The third system is HEARO®, a Swiss innovation which drills a narrow tunnel in the mastoid bone and through the facial recess (12, 13) to reach the RW through which the electrode is inserted (14). The HEARO® system received a CE mark in the year 2020. A new robotic system called Rosa®, another French innovation that offers robotic-controlled drilling of the mastoid and electrode insertion, has been recently evaluated for safety and accuracy in live patients (15). RoboJig is a German innovation currently under development. The robot drills a narrow tunnel in the mastoid guided by a jig that is developed on-site and is based on the patient's specific anatomy. It includes an automated insertion tool for the electrode (16). Recent reports in a series of patients demonstrate the clinical feasibility and effectiveness of these robotic systems in accommodating various CI electrode variants (14, 15, 17, 18).

The aim of RACIS is to eliminate or minimize intracochlear trauma during electrode insertion. Several electrode array insertion complications with a negative influence on post-operative outcomes have been reported after manual insertion. These include electrode tip fold over (ETFO) (19), electrode array scalar deviation (ESD) (20), and electrode array migration (EM) or slippage (21). A recent report on the application of RACIS also included one of the electrode-related complications mentioned above (17). Electrode variants that are currently available can be classified as either pre-shaped or straight electrode types (22). *Up-to-date* knowledge of the literature on the rate of these various electrode insertion complications by electrode type could facilitate electrode array selection for RACIS and for manual insertion. Therefore, this article is aimed to provide a systematic literature review on electrode-related insertion complications for both pre-shaped and straight electrodes.

## Methods

### Study Design

Following the recommendations of the Preferred Reporting Items for Systematic Reviews and Meta-Analysis (PRISMA) (23), the literature was systematically reviewed to establish the rate of ETFO, ESD, and EM for both pre-shaped and straight electrodes.

### Search Strategy and Study Selection

To perform the systematic literature review, a search for articles in PubMed, Scopus, Cochrane, and Web of Science was carried out using appropriate search terms (as listed in Table 1) by the first two authors PVH and PR. Articles published up to October 31, 2021 in English and German languages were considered for analysis. In addition, a manual search for relevant literature reviews and random checking of PubMed and Google Scholar were conducted using pertinent key terms. The first two authors independently screened titles and abstracts to select potential full-text articles according to the inclusion criteria. Exclusion criteria included review articles, surgical methodological studies, studies in languages other than English and German, studies using other approaches than through the posterior tympanotomy, and studies on auditory brain stem implants.

**Table 1:** Search terms used in the identification of relevant literature to perform the systematic literature review.

**Electrode insertion related complications**	**Search terms**
Electrode tip fold over (ETFO)	Cochlear implant electrode tip fold over or cochlear implant electrode tip roll over.
Electrode scalar deviation (ESD)	Cochlear implant electrode scalar deviation or cochlear implant electrode scalar location or cochlear implant electrode scalar position.
Electrode migration (EM)	Cochlear implant electrode-migration

### Data Extraction

A template in Microsoft Excel (www.microsoft.com/en-us/microsoft-365/excel) was created to record the extracted data, i.e., the first author of the study, study type, analyzing methods, the total number of ears implanted with CI, the number of ears implanted with each type of electrode, and, finally, the number of insertions with ETFO, ESD, and EM per electrode type.

### Data Analysis and Statistics

The rate of ETFO, ESD, and EM was calculated by dividing the number of ears with the associated issue by the total number of ears implanted with a specific type of electrode. Significance was calculated with the test for the difference of 2 proportions and 95% CIs, both implemented in MiniTab® (© 2019 Minitab, LLC, State College, PA, USA).

### Risk of Bias Assessment

The risk of bias was independently assessed by the third and the last authors (LL and JG). Included studies were assessed using the Risk of Bias in Non-randomized Studies of Interventions (ROBINS-I) tool (24). This tool contains seven items judging the risk of bias due to confounding, study participant selection, classification of interventions, deviations from intended intervention, missing data, measurement of outcomes, and selection of reported results. Each of the seven items in included studies was judged low, moderate, or high risk. Inner ear malformation was considered as the one of the confounding factors for ETFO, whereas electrode type was considered as the confounding factor for ESD and EM. Results of risk of bias assessment were graphically summarized using Microsoft Excel (https://www.microsoft.com/en-us/microsoft-365/excel).

## Results

### Search Results

**Figure 1:**
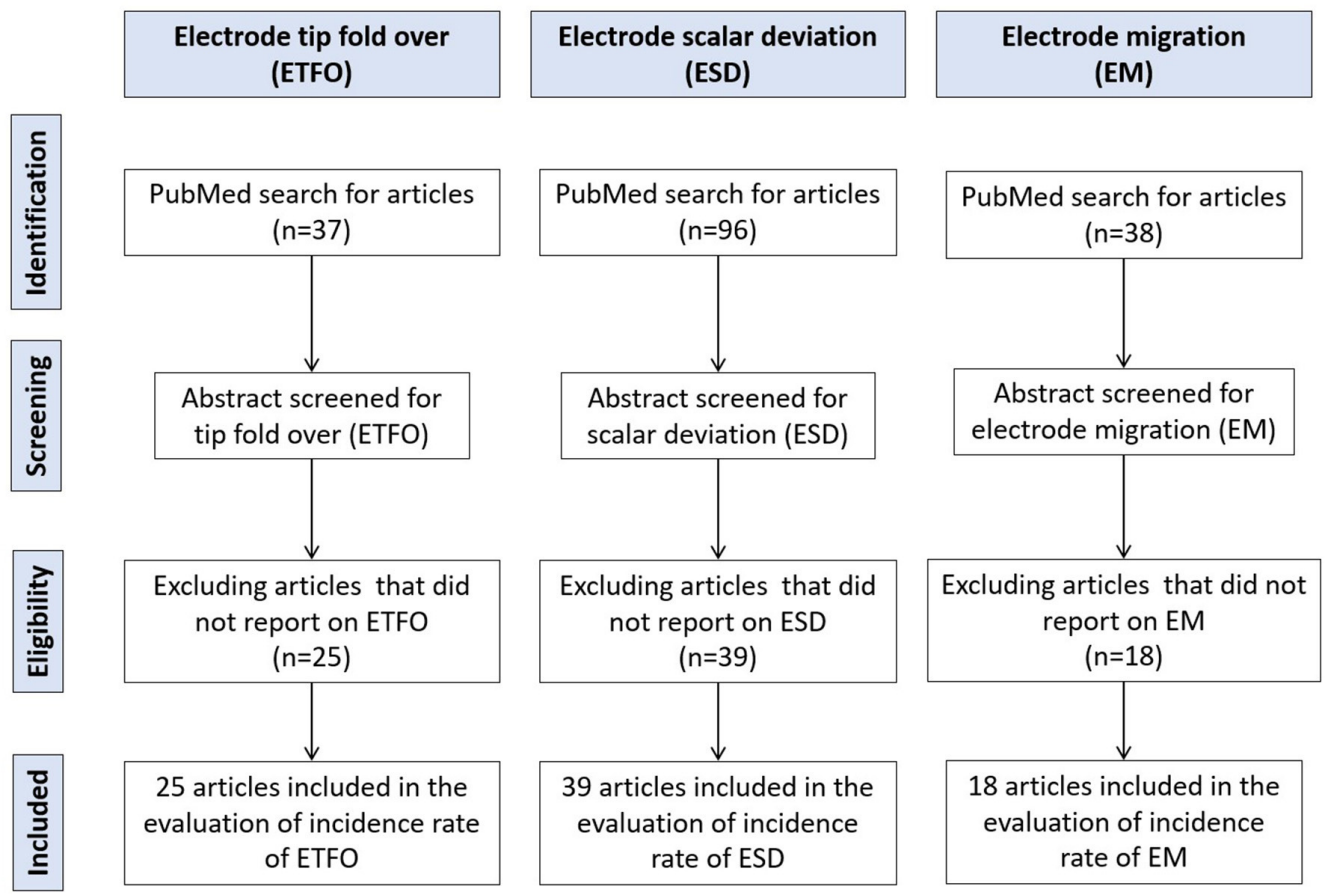
Literature review process utilizing the Preferred Reporting Items for Systematic Reviews and Meta-Analyses (PRISMA) guidelines.

Figure 1 details the systematic literature review process followed in the identification of relevant articles. A total of 37 articles on ETFO, 96 articles on ESD, and 38 articles on EM were identified using the search terms. After a thorough review of the abstract for search terms, 25 articles on ETFO, 39 articles on ESD, and 18 articles on EM were included in the evaluation of incidence rate.

### Risk of Bias

#### ROBINS-I—Risk of Bias Assessment

**Figure 2:**
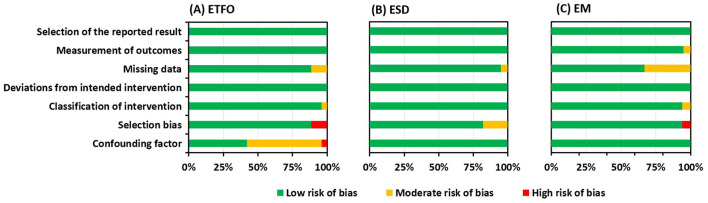
Risk of bias graph: review authors' judgment presented as percentages across all included studies about each risk of bias item for electrode tip fold over (ETFO) **(A)**, electrode scalar deviation (ESD) **(B)**, and electrode migration (EM) **(C)**.

The risk of bias assessment using the ROBINS-I tool is summarized in Figure 2. The majority of the studies included had a noticeable lower risk of bias as represented by green bars. All the studies identified specifically under all three electrode insertion complications had a low risk of bias for the reported result and for deviations from the intended intervention.

#### Confounding Bias

For the ETFO, cystic ear anatomy was considered a confounding factor, because cystic cochlear anatomy increases the chances of ETFO. Fourteen studies out of 25 did not reveal if the images of the selected patients were analyzed for any degree of anatomical deviation from the normal anatomy, suggesting those studies had a moderate risk of bias (studies 29–32, 34, 36–38, 40, 41, 43, 45, and 46). One study included the patients with the inner ear malformations and was graded with a high risk of bias (study 47). The type of electrode was taken as the confounding factor for ESD and EM. Electrode stiffness could increase the chances for ESD, and the pre-curved shape of the electrode would hook around the modiolar wall offering a natural fixation and minimizing the chances of EM. All the studies identified within ESD and EM issues reported the electrode type, suggesting a low risk of bias. The different sites and techniques of entering the ST, e.g., Cochleostomy (Coch) or RW, were not taken into account as a confounding factor.

#### Selection Bias

Selection bias mainly concerns how the patients were selected in the identified studies. Case series were graded with a high risk of bias whereas patients selected for a specific electrode type within a certain time period were thought to have a low risk of bias. Three out of 25 studies (studies 19, 43, 45) and 1 out of 18 studies (study 77) within ETFO and EM issues, respectively, were graded as having a high risk of bias. Seven out of 39 studies within the ESD issue were assigned a moderate risk of bias (studies 54, 57, 65-).

#### Missing Data Bias

Missing data bias becomes a concern if the type of electrode used is not specified for cases with electrode complications. Three studies within ETFO (Appendix 1: studies 7, 17, and 24), two studies within ESD (Appendix 2: studies 3 and 35), and 6 studies within EM issues (Appendix 3: studies 8, 11, 13–15, and 18) did not provide clear information on the electrode type and hence were considered to have a moderate risk of bias.

### Study Results

#### Electrode Tip Fold Over

Table 2 lists the 25 articles that reported on ETFO include the number of cases implanted and the type of electrode. Intra-operative or post-operative imaging was used in the identification of ETFO. A total of 5,042 ears were reported and after excluding the studies that did not specify the electrode type, 2,335 ears were taken for the evaluation. These 25 articles covered a total of 1,559 implantations with pre-shaped electrodes and 776 with straight electrodes. Eighty-four out of 1,559 ears implanted with a pre-shaped electrode, irrespective of CI brand, were associated with ETFO, an incidence rate (±95% CI) of 5.38% (4.4–6.6%). For the straight electrodes, irrespective of the CI brand, a rate (±95% CI) of only 0.51% (0.1–1.3%) was identified. The difference in rate between the pre-shaped and the straight electrode is highly significant (*p* < 0.001).

**Table 2:** Twenty-five articles reporting on electrode tip fold-over.

**Study/type**	**No. of cases taken for analysis/method**	**No. of electrode per type/brand**			**No. of cases reported tip fold-over**	
		**A**	**B**	**C**	**Pre-shaped**	**Straight**
Högerle et al. (25)/R	378 (Post-op x-ray)	–	–	FL (378)	–	0
Klabbers et al. (26)/P	25 (Intra-operative fluoroscopy)	SM (25)	–	–	3	–
Müller et al. (27)/R	108 (Spread of excitation/Intra-operative fluoroscopy)	SM (7), CA (87), SS (14)	–	–	CA (2), SM (2),	SS (1)
Durakovic et al. (28)/R	326 (Intra-operative x-rays)	SM (326)	–	–	23	–
Shaul et al. (29)/P	120 (Intra-operative x-ray)	SM (120)	–	–	8	–
Dimak et al. (30)/R	84 (Post-op x-ray)	SM (94)	–	–	3	–
Labadie et al. (31)/R	175 (Intra-operative imaging)	No info on brand segments: Straight electrodes (86); Pre-curved electrodes (89)			4 (SM) (not included in the analysis)	
Heutink et al. (32)/P	23 (Intra-operative fluoroscopy)	SM (23)	–	–	1	–
Garaycochea et al. (33)/R	19 (Intra-operative fluoroscopy)	SM (19)	–	–	3	–
Mittmann et al. (34)/R	85 (Flat-panel CT)	SM (85)	–	–	4	-
Iso-Mustajärvi et al. (35)/R	18 (Cone beam CT)	SM (18)	–	–	0	-
McJunkin et al. (36)/R	117 (Intra-op x-ray)	SM	–	–	9	–
Friedmann et al. (37)/R	237 (Intra-op x-ray)	SM (237)	–	–	11	–
Serrano et al. (38)/R	40 (Intra/Post-op x-ray)	SM (40)	–	–	2	–
Timm et al. (39)/R	275 (Post-op CT)	–	–	275 (F28, F24, F20, F16)	–	0
Sipari et al. (40)/R	23 (Post-op CBCT)	–	MS (23)	–	2	–
Gabrielpillai et al. (41)/R	1,722 (Post-op x-ray)	No info on brand segments			CA (7), SM (6), SS (2) (not included in the analysis)	
Jia et al. (42)/R	65 (Intra-op CBCT) (Contains 3 electrodes from Oticon)	CA (12), SM (1), SS (31)	1J (2), MS (3)	F28 (13)	SM (1)	–
Sabban et al. (19)/R	2 (x-ray & CT)	–	MS	–	2	–
Garaycochea et al. (43)/R	1 (Intra-op fluoroscopy)	SM	–	–	1 (100%)	–
Aschendorff et al. (44)/R	45 (Post-op CBCT)	SM	–	–	2 cases. 1st case corrected in the same surgery. 2nd case underwent revision surgery	–
Zuniga et al. (45)/R	303 (Post-op CT)	CA, SS	MS, 1J	–	CA (3), [MS (1), SS (1) and 1J (1)]	
		No info on brand segments			(not included in the analysis)	
Fischer et al. (46)/R	63 (Post-op CBCT)	–	–	F24, F28, Std	–	1
Dirr et al. (47)/R	215 (Post-op x-ray)	CA, SS	–	Std, M, S, FL, F28	FL (2)	
		No info on brand segments			(not included in the analysis)	
Cosetti et al. (48)/R	277 (Intra-op x-ray)	CA	–	–	5	–
Total	5,042				110 (102 pre-shaped electrodes + 8 straight electrodes)	
Total, after excluding four studies that did not specify number per electrode type	2,335	Pre-shaped (1,559), Straight (776)			Pre-shaped (84), Straight (4)	

*R, retrospective; P, prospective; SM, Slim-Modiolar; CA, contour advance; MS, mid-scala; SS, slim straight; Std, standard; M, medium; S, compressed; FL, FLEX SOFT, F28: FLEX^28^; F24, FLEX^24^; F20: FLEX^20^; F16: FLEX^16^; CBCT, cone-beam CT. Studies that are shaded in gray were not included in the analysis due to the non-availability of information on the CI brand*.

### Electrode Scalar Deviation

Table 3 lists the 39 articles which reported ESD along with the number of ears implanted and the type of electrode. Different modalities, such as electrocochleography (EcochG), intra-operative fluoroscopy or CT, and post-operative CT imaging, were used to detect the ESD. A total of 3,073 ears (1,983 pre-shaped and 1,090 straight electrodes) were included for further analysis after excluding studies that did not specify the electrode type. Out of 1,983 ears implanted with pre-shaped electrodes, irrespective of manufacturer, ESD was reported in 567 ears yielding a rate (±95% CI) of 28.6% (26.6–30.6%). Out of 1,090 ears implanted with straight electrodes, irrespective of manufacturer, ESD was reported in 120 ears yielding a rate (±95% CI) of 11% (9.2–13.0%). The difference in rate between the pre-shaped and the straight electrode is highly significant (*p* < 0.001).

**Table 3:** Thirty-eight studies reported on electrode scalar deviation.

**Study/type**	**No. of analyzed cases**	**Analyzing method**	**No. of electrode from type/brand**			**No. of cases reported with scalar deviation**	
			**A**	**B**	**C**	**Pre-shaped**	**Straight**
Riemann et al. (49)/P	20	3T MRI	–	MS (5), SJ (5)	F28 (10)	1	0
Liebcher et al. (50)/R	255	Post-op CT	CA (99), SM (156)	–	–	32 (CA), 8 (SM)	—
Heutink et al. (51)/R	129	Post-op CT	CA (85), SS (44)	–	–	20 (CA)	SS (18)
Ketterer et al. (52)/R	201	Post-op CBCT	–	–	F24 (28), F26 (15), F28 (139), FL (19)	–	F24 (1), F28 (6), FL (5)
Lenarz et al. (53)/R	20	Post-op CBCT	–	SJ (20)	–	0	–
Durakovic et al. (28)/R	76	Post-op CT	SM (76)	–	–	5	–
Morrel et al. (54)/P	177	Post-op CBCT	SS (46)	1J/SJ (39)	F24 (8), F28 (52), Std (32)	–	39
Nassiri et al. (55)/R	24	Intra-op CT	SM (24)	–	–	1	–
Heutink et al. (32)/P	23	Post-op CT	SM (23)	–	–	8	–
Iso-Mustajärvi et al. (35)/R	18	Post-op CBCT	SM (18)	–	–	0	–
Riggs et al. (56)/P	21	Post-op CT/EcochG	–	MS (21)	–	7	–
Chakravorti et al. (57)/R	220	Post-op CT	CA (89), SS (20), ST (11)	MS (21), 1J (29)	F24 (3), F28 (22), M (1), Std (24)	45	11
Yamamoto et al. (58)/R	58	Intraoperative CT	CA/C (30), SS (12)	1 (MS)	F24 (3), F28 (12)	16	7
Shaul et al. (59)/P	110	Post-op CBCT	CA (92), SM (18)	–	–	18	–
Sipari et al. (40)/R	23	Post-op CT	–	MS (23)	–	5	–
Koka et al. (60)/P	32	Post-op CT/EcochG	–	MS (32)	–	7	–
Jia et al. (42)/R	65	Intra-op CT	CA (12), SM (1), SS (31)	1J (2), MS (3)	F28 (16),	1	–
McJunkin et al. (36)/R	23	Post-op CT	SM (23)	–	–	6	–
Ketterer et al. (61)/R	368	Post-op CBCT	CA (368)	–	–	118	–
An et al. (62)/R	26	Post-op CT	SS (5)	–	F28 (21)	–	F28 (1), SS (1)
Aschendorff et al. (44)/P	45	Post-op CBCT	SM (45)	–	–	0	–
O'Connell et al. (63)/R	48	Post-op CT	–	–	F24, F28, Std (48)	-	0
O'Connell et al. (64)/P	18	EcochG/Post-op CT	–	MS (18)	–	6	–
Mittmann et al. (65)/R	50	NRT/Post-op CT	SS (50)	–	–	-	SS (2)
Lathuilliere et al. (66)/P	24	Post-op CBCT	CA (24),	–	–	3	–
O'Connell et al. (67)/R	56	Post-op CT	CA (36), SS (20)	–	–	19	SS (2)
O' Connell et al. (68)/R	220	Post-op CT	CA (115), SS (19),	1J (21), MS (14)	F28 (28), Std (17), F24 (4) & M (2)	67	F (4)
Wanna et al. (69)/P	45	Post-op CT	CA (15)	MS (3)		5	1J and SSS (2)
			SS, 1J & F collectively (27=9 each)				
Nordfalk et al. (70)/R	39	Post-op CT	–	–	F28 (18), FL (17), F24 (4)	–	F (0)
Mittmann et al. (71)/R	23	NRT/Post-op CT	CA (23)	–	–	6	–
Mittmann et al. (71)/R	85	NRT/Post-op CT	CA (85)	–	–	16	–
Boyer et al. (72)/n/a	61	Post-op CBCT	CA (31),	–	FL, F28, F24, Std (30)	8	F (0), Std (1)
Fischer et al. (46)/R	63	Post-op CBCT	–	–	F28 (40), F24 (2), FL (7), Std (14)	–	F28 (5)
Wanna et al. (73)/P	116	Post-op CT	CA (35), MS (34)		(47) LW from all 3 CI brands (15, 15, 17)	29	All LW (5)
Dirr et al. (47)/R	215	Post-op x-ray	107		108	–	F (1)
Nordfalk et al. (74)/R	13	Post-op CT	CA (7)	1J (3)	Std (2), F24 (1)	3 (CA)	Std (1), 1J (1)
Aschendorff et al. (75)/R	223	Post-op CT	C (21), CA (202)	–	–	19 (C), 70 (CA)	–
Wanna et al. (73)/R	32	Post-op CT	20	10	2	11	F (0)
Lane et al. (76)/R	23	Post-op CT	C/CA (13)	H (1)	–	6 (C)	LW (7)
			LW electrodes from brand A (5) & B (4)				
Total (excluding Dirr et. al)	3,073		2,073	333	667	567	120
			Pre-shaped (1,983) Straight (1,090)			Pre-shaped (567) Straight (120)	

*R, retrospective; P, progressive; n/a, non-availability of data; SM, Slim-Modiolar; CA, contour advance; MS, mid-scala; SS, slim straight; SJ, Slim J; Std, standard; M, medium; S, compressed; FL, FLEX SOFT, F28: FLEX^28^; F24, FLEX^24^; F20: FLEX^20^; F16: FLEX^16^; NRT, neural response telemetry; EcochG, electrocochleography. A study that is shaded in gray was not included in the analysis due to the non-availability of information on the CI brand*.

### Electrode Migration

Table 4 lists the 18 articles which reported on EM. Post-operative imaging was used in the identification of EM. A total of 5,795 ears implanted with CI were identified from the literature search. After excluding those studies that did not specify the electrode type, a total of 3,195 ears were taken for analysis. Pre-shaped electrodes were implanted in 1,327 ears and straight electrodes were implanted in 1,868 ears. EM was identified in 61 ears implanted with straight electrodes, an incidence rate (±95% CI) of 3.2% (2.5–3.95%). For pre-shaped electrodes, only 7 ears were identified with EM, a rate (±95% CI) of around 0.53% (0.2–1.1%). The difference in proportion between the pre-shaped and the straight, electrode is highly significant (*p* < 0.001).

**Table 4:** Eighteen studies reported on electrode migration.

**Study/type**	**No. of analyzed cases**	**Analyzing method**	**No. of electrode from type**		**No. of cases reported with electrode migration**	
			**Pre-shape**	**Straight**	**Pre-shaped**	**Straight**
Ozer et al. (77)/R	149	Post-op CT	–	149	–	1
Chan et al. (78)/R	1	Post-op x-ray and CT	–	1	–	1
Mitzlaff et al. (79)/R	560	Post-op CT	414	146	–	6
Leinung et al. (80)/R	1,603	Post-op x-ray and CT	772	831	–	17
Rajan et al. (81)/R	56	Not mentioned	–	56	–	1
Celik et al. (82)/R	245	Post-op x-ray	Not specified	Not specified	–	1
Rader et al. (83)/R	270	Post-op CBCT	–	270	–	10
Patnaik et al. (84)/R	534	Post-op HRCT	Not specified	Not specified	–	2
Mittmann et al. (71)/R	54	Post-op CT	54	–	7	–
Dietz et al. (21)/R	201	Post-op CBCT	64	137	–	12
Jeppesen et al. (85)/R	308	Post-op CT	Not specified	Not specified	–	4
van der Marel et al. (86)/R	35	Post-op CT	–	35	–	10
Lavinsky-Wolff et al. (87)/R	75	Post-op X-ray	Not specified	Not specified	–	2
Brown et al. (88)/R	806	Post-op CT	Not specified	Not specified	–	4
Connell et al. (89)/R	580	Post-op CT	Not specified	Not specified	–	2
Green et al. (90)/R	239	Post-op imaging	23	216	–	3
Roland Jr et al. (91)/P	27	Post-op x-ray	–	27	–	0
de Long et al. (92)/R	52	Post-op imaging	Not specified	Not specified	–	0
Total	5,795				7	83
Excluding studies that did not specify the electrode type	3,195		1,327	1,868	7	61

*R, Retrospective; P, Progressive; CBCT, Cone-Beam Computerized Tomography*.

## Discussion

### Summary of Evidence

The aim of this literature review was to determine the type of electrode best suited to minimize deleterious complications for use in RACIS and in conventional CI surgery. This systematic literature review yielded a total of 82 studies covering a total of 8,603 CI procedures, which met within the inclusion criteria. This review specifically sought to establish the incidence of ETFO, ESD, and EM for both pre-shaped and straight electrodes. A total of 4,869 ears implanted with pre-shaped and 3,734 ears implanted with straight electrodes were identified from the search. The high number of CI procedures (8,603) in total allowed us to compare the rate of electrode insertion complications between the two electrode types, which are of value for RACIS and conventional CI surgery.

### Electrode Tip Fold Over

**Figure 3:**
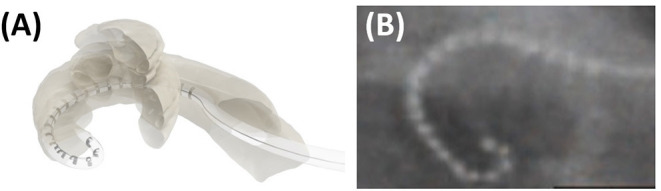
Cartoon picture demonstrating how an electrode tip fold over would look like **(A)**. Post-operative plain film x-ray showing electrode tip fold-over in a patient case **(B)**. Reproduced by permission of Wolters Kluwer Health Inc. (Appendix 1—reference 17).

An ETFO occurs when the tip of the electrode gets stuck in the ST and, on further insertion, the tip bends back on itself as shown in Figures 3A,B. This could provoke short circuiting between the apical electrode contacts and can result in pitch confusion and perversion. Moreover, it may damage the basilar membrane leading afterward to fibrosis, hydrops, and ossification (93).

Electrode tip fold over is associated in most cases with varying degrees of decreased speech understanding and, in several series, co-stimulation of the facial nerve and dizziness was reported. The speech understanding may be as low as 20% with a Bamford-Kowal-Bench (BKB) speech test in quiet (38) or with hearing in noise test (HINT) (19) up to a reported case with preserved residual hearing and one with 80% speech in Quite (45). Revision operations or deselecting the involved electrodes increased in most cases the speech and solved the complaints of facial co-stimulation and dizziness (19, 41, 45). Intra-operative imaging is one possible means of detecting the ETFO during surgery in which case it can be corrected as part of the initial surgery, as it has been suggested by several clinicians (19, 28, 43).

This literature review demonstrates that ETFO is more commonly associated with pre-shaped electrodes (rate of 5.3%) than with straight electrodes (0.5%). Based on reasons cited in the literature, the higher prevalence in pre-shaped electrodes could be due to any of the following factors: the pre-mature pulling of the stylet/polymer sheath, the orientation of the electrode contacts away from the modiolar wall during insertion, variations in the size and shape of the cochlea, and variations in the length of the straight portion of the basal turn. The shape of the electrode tip (conical/pointed geometry) is another design-related factor that could influence the incidence of ETFO issues (25, 94, 95).

Once the electrode is inserted inside the ST, it follows its own path, and currently, there are no steerable electrodes available. Experimental work on cadaveric temporal bones demonstrated that robotic insertion could reduce intracochlear trauma by applying a constant insertion speed in an optimized axis (96). Hence, it is to be expected that the application of RACIS would lead to less traumatic insertions. However, there is no evidence yet that better control of insertion speed, as offered by some systems, or an insertion more axial to the basal part of ST, as offered by other robotic systems would decrease the rate of ETFO with pre-shaped electrodes. Testing in the future will determine whether the implementation of haptic pressure feedback might detect a tip getting stuck.

### Electrode Scalar Deviation

**Figure 4:**
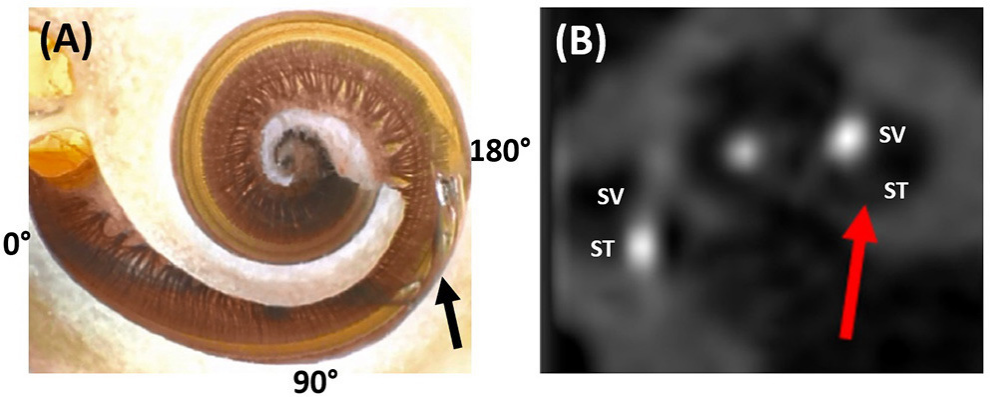
Dissected cochlear sample showing the electrode tip of a pre-shaped electrode penetrating the spiral ligament from the scala tympani (ST) and translocating to scala vestibuli (SV) **(A)**. Image courtesy of Prof. Peter Roland from Southwestern University, TX, USA. Post-operative CT image slice showing part of the electrode in the SV as pointed by the red arrow **(B)**. Reproduced by permission of Elsevier B.V (Appendix 2—reference 2).

Electrode scalar deviation means that the electrode which is inserted into the ST through an RW or Coch approach perforates the basilar membrane and a number of apical electrodes end up in SV. ESD is by far the most frequent serious complication. This occurs mainly between 90 and 180° of angular insertion depth, causing a scalar deviation as pointed by a black arrow in Figure 4A and a red arrow in Figure 4B.

Electrode scalar deviation is associated with fibrous tissue growth and osteo-neogenesis with the cochlea. Most importantly, ESD has been associated with irreversible degeneration of neuronal cells as detected from the histological evaluation of cadaveric temporal bones from patients who in life had undergone CI (97). Breaching the basilar membrane and allowing the mixture of perilymph and endolymph can result in the loss of any residual hearing.

Electrode scalar deviation is associated with poorer hearing outcomes when compared to patients with no ESD (20, 67, 68). Jwair et al. through a meta-analysis on ESD identified six studies that compared post-operative speech perception scores between post-lingually adult CI recipients with and without ESD. They concluded that ESD was negatively associated with speech perception scores (weighted mean 41%) compared to full ST placement (weighted mean 55%) (20). O' Connell et al. in 2016 reported the rates of 22.4 and 55% ESD with contour advance (CA, pre-shaped) and mid-scala (MS; pre-shaped) electrodes, respectively, and the ESD was associated with a 12% decrease in consonant-nucleus-consonant (CNC) score (67). O'Connell et al. in 2016 through a literature review covering 6 studies reported that ST insertions are associated with better speech performance when compared to patients with SD. They further reported that the SD affects the low-frequency residual hearing of patients negatively (68).

Electrode scalar deviation is more frequent with pre-shaped electrodes (rate of 28.7%) than with straight electrodes (rate of 11%) in this literature review. The reasons for the higher rate might be explained as follows: (1) due to the variation in cochlear size, shape, and the length of the straight portion of the cochlear basal turn, the standard insertion depth to which the straightened pre-shaped electrode should be inserted inside the cochlea prior to stylet rod/polymer sheath removal could already be deep enough to penetrate the spiral ligament. (2) Orientation of the contact pads of the pre-shaped electrode away from the modiolus wall and facing the basilar membrane/spiral ligament may cause the pre-shaped electrode to curl upward (rather than laterally around the modiolus) when the stylet rod/polymer sheath is retracted. This would cause the tip to penetrate the osseous spiral lamina or basilar membrane. In contrast, the straight electrode has the flexibility to bend in all directions, making it far less traumatic even if the electrode contacts are oriented away from the modiolar wall (94).

The different sites and techniques of entering the ST, e.g., Coch, RW, or extended RW (ERW), approach may also have an influence on ESD. Mainly CI studies in case of residual hearing addressed this issue. Although the approach could not be analyzed as a confounding factor, it deserves special attention. The first multicenter studies that reported atraumatic electrode insertions used a Coch approach (98) and later studies with long-term follow-up could not demonstrate a difference between RW and Coch (99).

Studies focusing on ESD have demonstrated that electrode insertion through RW is associated with a lower incidence of ESD, compared to a Coch approach (46, 73). A consensus publication on atraumatic insertion strongly advocated the RW approach (2). A histopathological study by Ishiyama et al. analyzed the temporal bones of CI patients who in life underwent CI surgery with either an RW or a Coch approach revealed that although insertion through a standard promontory Coch resulted in hydrops and fibrosis in both the ST and SV in the majority of subjects, RW insertions did not (100). Hence, RACIS aims for minimal traumatic inner ear access at the level of the RW in the case of normal anatomy (101).

Cadaveric temporal bone experiments show that, in particular, the occurrence of ESD is decreased in motorized co-axial insertion with a slow steady speed (102). Yet, even in the limited series of RACIS, ESD has been reported with pre-shaped electrodes (17). With straight electrodes, RACIS can better manage co-axial insertion into the ST, minimizing damage to the scalar walls. Indeed, studies have shown that the orientation of insertion with a robotic system reduces both the error and the variability of the alignment to a defined optimal axis that it is significantly better compared with a manual insertion, even with experienced surgeons (102, 103). The detection of premature electrode contact with the basilar membrane is expected to improve when intra-operative evoked potentials can be reliably measured (64, 104, 105) with advanced intra-operative imaging (106).

### Electrode Migration

**Figure 5:**
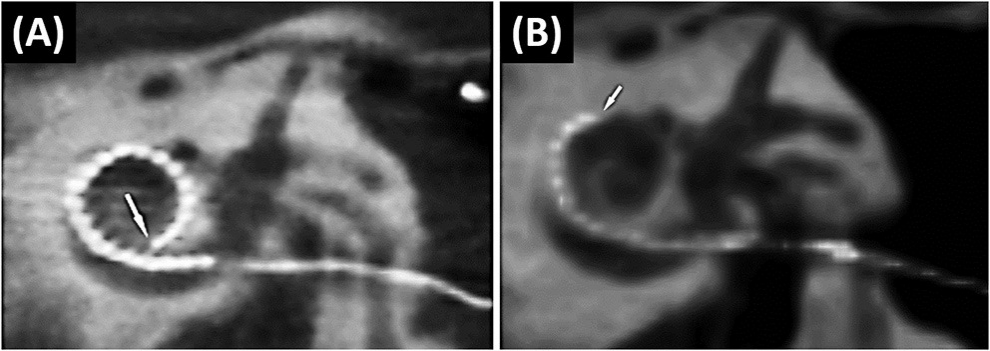
The immediate post-operative CT scan shows a fully inserted Cochlear Slim Straight array (CI422) with an insertion angle of 390°**(A)**. The follow-up scan shows a substantially retracted electrode with six extra-cochlear electrodes and an insertion angle of 210°. The arrow points to the tip of the electrode (21) **(B)**. Reproduced by permission of Springer Nature.

In the case of EM, the electrode retracts from its original intracochlear position. This results in the partial displacement of some electrode contacts outside the cochlea. Although, it is believed not to occur often in the opinion of several experienced surgeons, EM is underreported (21). Figure 5A shows a fully inserted electrode immediately post-op. A follow-up scan, however, shows that the electrode array has retracted out of the cochlea (Figure 5B).

**Figure 6:**
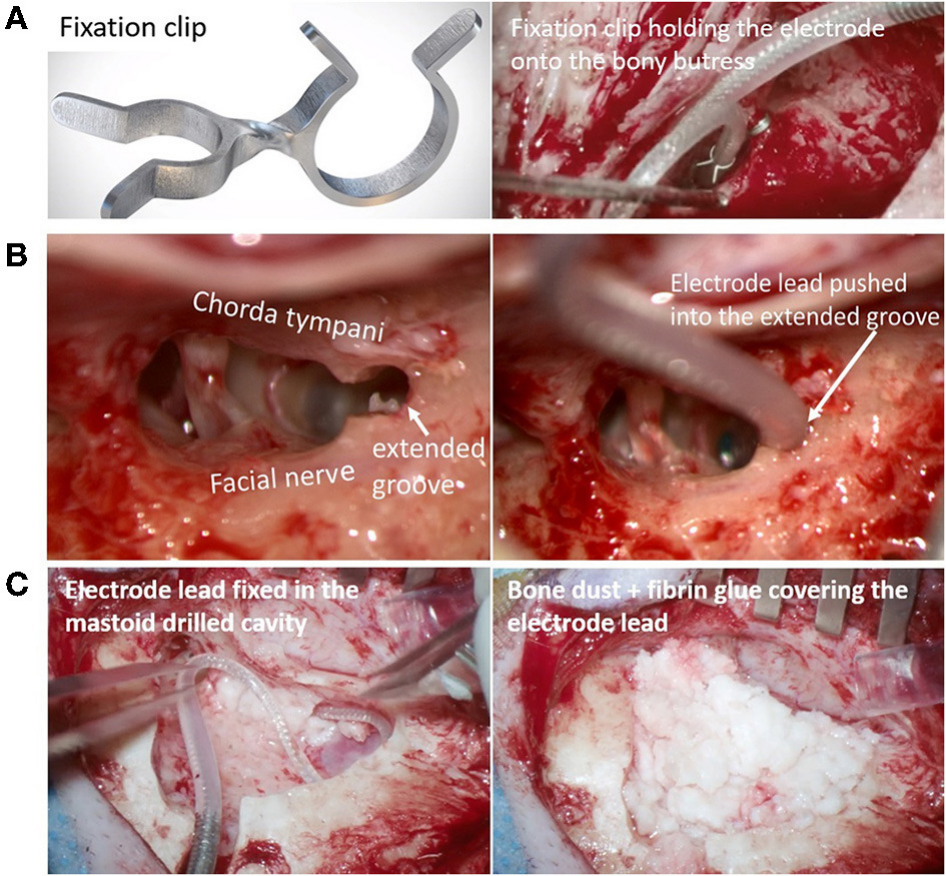
Fixation clip holding the electrode lead onto the bony-buttress of the middle ear space [**(A)** Image courtesy of Joachim Müller, Munich, Germany]. Electrode lead pushed into the extended groove between the facial nerve and chorda tympani [**(B)** Image courtesy of Timo Stöver, Frankfurt, Germany]. The electrode lead is fixed in the posterior tympanotomy [**(C)**-left] and the excess electrode lead coil within the undercut cortex is covered with bone dust mixed with fibrin glue [**(C)**-right- Image courtesy of Paul Van de Heyning, Antwerp, Belgium].

Electrode migration can occur during the closing phase of surgery, immediately post-operatively or later on. EM can result in increased electrode impedances and deterioration of speech recognition scores (21). Depending on the number of extra-cochlear electrodes and the associated impact on hearing, revision surgery to reinsert the electrode into the cochlea may be undertaken. The reason for EM with a straight electrode is believed to be the spring-back force stored in the excess electrode lead coiled in the mastoid drilled cavity. Even a slight relaxation in the coiled electrode lead in the mastoid cavity due to the patient's activity or natural mastoid growth (107) could potentially pull the electrode array out of the cochlea. A possible solution is the electrode lead fixation clip, as shown in Figure 6A, that could minimize/prevent electrode movement and retraction (108). Alternatively, a gentle groove between the facial nerve and chorda tympani (as shown in Figure 6B) into which the electrode lead is placed has limited the movement of the electrode lead (109). Fixing the electrode with bone dust mixed with fibrin glue (as shown in Figure 6C) is advocated by some surgeons (110).

Electrode migration occurs more commonly with the straight electrodes (rate of 3.2%) than with pre-shaped electrodes (rate of 0.53%). EM out of the cochlea is usually not associated with pre-shaped electrodes because the curved electrode array acts like a hook around the modiolus which provides 5–10 times the holding force needed to extract the electrode from the cochlea compared to straight electrodes (71). Nevertheless, fixating the electrode is advocated for all types of electrodes and not only to prevent EM but also to reduce fatigue electrode wire breakage due to electrode micromovements.

Robotic systems, such as RobOtol® and iotaSOFT®, that insert the electrode through the classic CI approach with an open mastoid and posterior tympanotomy have the same options as that of manual electrode insertion in stabilizing the electrode regardless of the electrode types. Robotic systems, such as HEARO®, Rosa®, which drill a narrow tunnel (direct cochlear access) from the cortex to the cochlea, need an alternate solution for stabilizing the electrode. EM has not been reported in the limited series of patients operated on who have had robotic insertions (12–15, 17, 18). Although the narrow tunnel approach itself provides some stabilization and the absence of coiled excess electrode lead in the mastoid cavity minimizes the EMs, caution is needed in fixing the electrode in the tunnel, which might be accomplished, for example with bone paté.

### RACIS and Electrode Type

As the main goal of RACIS is to be less traumatic, this literature review favors the use of straight electrodes due to the significantly lower incidence of ETFO and ESD. ESD often results in irreversible an intra-cochlear injury that permanently degrades hearing outcomes. ETFO and EM, however, are generally correctable and do not result in permanent cochlear damage. Therefore, minimizing the risk of scalar translocations should be a high priority, none-the-less, special care has to be taken to avoid EM when using straight electrodes. This is in line with the conclusions of Jwair et al., ‘if one aims to minimize clinically relevant intracochlear trauma, lateral wall arrays would be the preferred option for cochlear implantation’ (20).

It is to be hoped that RACIS could further decrease the occurrence of these complications when motorized insertion, with a slow steady speed, is combined with directional control in all three planes to realize an optimized alignment with the ST. Robotic systems have proved to be superior in controlling both speed and directionality. Doudi et al. recently reported from their clinical study comparing 40 CI patients with manual insertion with 20 CI patients with robotic insertion showed a less ESD for robotic insertion of straight electrode arrays when compared with manual insertion (111).

A study by Barriat et al. in 2021 reported complete hearing preservation with a mean loss of pure tone average for five frequencies of 13.60 ± 7.70 dB, and this was associated with a lower insertion speed of 0.88 ± 0.12 mm/s applied by RobOtol® (10). One must realize, however, that the anatomical course of the facial nerve prohibits a perfect co-axial approach to the ST. Animal studies have demonstrated that flexible electrodes are associated with less ESD, thereby, minimizing the hearing loss and intra-cochlear fibrosis (112).

These conclusions are based on a large number of cases taken for analysis from 82 studies. Due to the heterogeneous design of all these studies, a meta-analysis with a forest plot could not be made. This literature review focused on three deleterious complications affecting the hearing outcomes linked with the use of two types of electrodes. There are many factors that have an impact on the electrode choice in RACIS, such as electrode length, electrode stiffness (113), and electrode insertion path that includes both a direct tunnel approach and through posterior tympanotomy. While the electrode insertion through a posterior tympanotomy approach can handle any type of electrode, the direct tunnel approach can only handle straight electrodes. Electrode selection matching the cochlear anatomy, the cochlear duct length, and spiral ganglion cell body distribution (114–117) will prove beneficial when combined with a robotic-assisted electrode insertion and pre-planned computational insertion angles and electrode lengths of 16–34 mm.

### Strengths and Limitations

This Systematic Review (SR) provides a systematic evaluation that includes the risk of bias assessment of published evidence on the topic of ETFO, ESD, and EM that are associated with manual insertion of electrodes. The possibility of reducing electrode insertion complications through electrode design is of high relevance to healthcare providers and patients. The electrode insertion complications as reported in the identified articles were confirmed by visually looking at either intra-operative or post-operative images that decrease the overall bias with measurement of outcomes. We did this systematic review strictly following the PRISMA guidelines of reporting. Limitations include the bias in the studies identified mainly due to the risk of selection and confounding bias. Most of the studies identified were retrospective in nature.

## Conclusions

The design of the electrode influences the incidence of electrode insertion complications. The literature findings of the current study reveal that there is a higher incidence of ETFO and ESD associated with pre-shaped electrodes compared to straight electrodes. EM, on the other hand, occurs more often with straight lateral wall electrodes. *Ex vivo* experiments and clinical studies indicate that the application of robotic systems could optimize the electrode insertion characteristics thereby reducing the insertion-related issues. Robotic-assisted electrode insertion and manual insertion should be complemented with the straight electrode design that is associated with the least positioning complications.

## Data Availability Statement

The original contributions presented in the study are included in the article/supplementary material, further inquiries can be directed to the corresponding author/s.

## Author Contributions

PV and PR: study design, search for articles, review of articles, data extraction, and manuscript writing. LL and JG: quality assessment of the articles identified and manuscript writing. The remaining authors were involved in the study design, data analysis, manuscript editing, and overall discussion. All authors contributed to the article and approved the submitted version.

## Conflict of Interest

MK is the chief surgeon and Director of Madras ENT Research Foundation Pvt. LTD., which is the organization he founded and for which he is currently working for. The remaining authors declare that the research was conducted in the absence of any commercial or financial relationships that could be construed as a potential conflict of interest.

## Publisher's Note

All claims expressed in this article are solely those of the authors and do not necessarily represent those of their affiliated organizations, or those of the publisher, the editors and the reviewers. Any product that may be evaluated in this article, or claim that may be made by its manufacturer, is not guaranteed or endorsed by the publisher.
